# Methodological challenges in utilizing miRNAs as circulating biomarkers

**DOI:** 10.1111/jcmm.12236

**Published:** 2014-02-18

**Authors:** Leni Moldovan, Kara E Batte, Joanne Trgovcich, Jon Wisler, Clay B Marsh, Melissa Piper

**Affiliations:** a Division of Pulmonary, Allergy, Critical Care, Sleep Medicine, College of Medicine, The Ohio State UniversityColumbus, OH, USA; b Comprehensive Cancer Center, College of Medicine, The Ohio State UniversityColumbus, OH, USA; c The Department of Surgery, College of Medicine, The Ohio State UniversityColumbus, OH, USA

**Keywords:** biomarkers, microRNA, circulating microRNAs, plasma, serum, real-time PCR, profiling

## Abstract

MicroRNAs (miRNAs) have emerged as important regulators in the post-transcriptional control of gene expression. The discovery of their presence not only in tissues but also in extratissular fluids, including blood, urine and cerebro-spinal fluid, together with their changes in expression in various pathological conditions, has implicated these extracellular miRNAs as informative biomarkers of disease. However, exploiting miRNAs in this capacity requires methodological rigour. Here, we report several key procedural aspects of miRNA isolation from plasma and serum, as exemplified by research in cardiovascular and pulmonary diseases. We also highlight the advantages and disadvantages of various profiling methods to determine the expression levels of plasma-and serum-derived miRNAs. Attention to such methodological details is critical, as circulating miRNAs become diagnostic tools for various human diseases.

## Introduction

MicroRNAs (miRNAs) are small non-coding RNA sequences of about 22 nucleotides that are important post-transcriptional regulators of gene expression. They impact many if not most developmental [1] and homeostatic processes such as the immune response and metabolism [2, 3] and epigenetic processes as well [1, 4].

A plethora of studies in humans, complemented by animal models, demonstrate the importance of tissue miRNAs during developmental processes [5–11] and various disease pathologies [12–17]. Thus, significant changes of tissue miRNA ‘signatures' occur in various diseases, such as cancers [18, 19], cardiovascular disease (CVD)[21–26], diseases of the lung [27–31], kidney [16] and nervous system [32, 33]. However, routine biopsies from any organ for miRNA profiling are not a practical option; therefore, investigators are turning towards less invasive procedures, involving circulating miRNA biomarkers (for a review of practical issues, see [34]).

The presence of miRNA molecules in human plasma was concomitantly reported by several groups [35–38]. Once it became clear that some miRNAs were released from their cells of origin and could be captured in various extracellular fluids, numerous studies began investigating whether tissue-and disease-specific miRNA signatures were also reflected in fluids such as blood, urine, spinal fluid or saliva [39]. Extracellular miRNAs are remarkably stable in the circulation [35–37, 40]. As a result of cellular damage/tissue injury, such as in acute myocardial infarction (MI) [41, 42], atherosclerosis [44], non-small cell lung cancer [45], neurodegenerative diseases [46–49], skin fibrosis [50] and osteoarthritis [51] (Table 1), miRNA expression can change in the blood. Notably, this list is increasingly expanding. Furthermore, miRNA expression can change not only in the blood but in other body fluids as well (Table 1). Thus, circulating miRNAs are attractive candidates for disease monitoring to serve as valuable prognostic indicators of disease progression or resolution. It is predicted that changes in miRNA expression in body fluids occur earlier than conventional biomarkers. For instance, the markers of inflammation and repair in the cardiovascular and lung systems include troponin, C-reactive protein, chemokines and cytokines [52, 53]. However, by the time these proteins are detectable in the circulation, much of the tissue damage has already occurred, which makes it crucial that better biomarkers be uncovered for the early detection of diseases.

**Table 1:** Examples of circulating miRNAs detected in various human pathologies

MiRNAs differentially expressed	Condition	Sample type	Anticoagulant	Volume (ml)	Isolation method*	Spike-in/endogenous controls	Detection method	No. of miRNAs tested	Refs.
miRs-21,-155,-210	Diffuse large B-cell lymphoma	Serum	N/A	2	Trizol	miR-16	qRT-PCR†	3	[71]‡
N/A	Healthy donors	Plasma	Unknown	40	Trizol	None	qRT-PCR pre-amp	420	[36]§
let-7a, miRs-16,-92a,-122,-142-3p,-150	Healthy donors	Plasma (p), EV, Serum (s)	EDTA	30 (p) 10 (s)	miRNeasy; 5 vol Qiazol	cel-miRs	Microarray¶ qRT-PCR	375	[68]**
miRs-15b,-16	Prostate cancer	Plasma Serum	EDTA	10	mirVana PARIS	cel-miRs	qRT-PCR pre-amp	368	[37]††
miRs-16,-21,-24,-34b	Healthy donors	Plasma	EDTA	0.4	Tri-Reagent LS miRNeasy	cel-miR-39	qRT-PCR pre-amp	667	[72]‡‡
miRs-15b,-16,-24,-122	Healthy donors	Plasma Serum	EDTA	0.4	mirVana PARIS	cel-miRs	qRT-PCR	3	[73]§§
let-7b, miR-223	NSCLC	Plasma Serum	Heparin	0.1	Total RNA purification kit	cel-miRs	qRT-PCR	30	[74]§§
N/A	N/A	Plasma Serum	U	0.4	miRNeasy; 10 vol Qiazol	cel-miRs	qRT-PCR	N/A	[75]¶¶
miR-499	ACS; CHF	Plasma	EDTA	0.5	mirVana PARIS	Novel Synthetic miR	qRT-PCR	1	[41]
miRs-1,-133a,-133b,-208a,-208b,-499	ACS	Plasma	U	0.05	Master Pure RNA Purification Kit	cel-miR-54	qRT-PCR	6	[43]
miRs-1,-133	AMI	Plasma	Citrate	1	miRNeasy	U6 snRNA	qRT-PCR	2	[42]
miR-1	AMI‡‡	Serum	N/A	0.2	miRNAs Isolation Kit	None (absolute quantification)	qRT-PCR	1	[76]
miRs-1,-16, 133a,-208a,-499	AMI‡‡	Plasma	EDTA	0.1	TRI Reagent BD TR126	cel-miR-39	Microarray qRT-PCR	5	[77]
miRs-1,-122, 133a,-133b,-375,-499-5p	AMI‡‡	Plasma Serum	EDTA; thrombin	2.4	mirVana PARIS	miR-17-5p; median normalization	qRT-PCR	667	[52]
miRs-208b,-499	AMI, VM, DD; AHF	Plasma	Citrate; EDTA	0.1	mirVana PARIS	cel-miRs	qRT-PCR	6–12	[78]
miRs-1,-133a	AMI‡‡	Serum	N/A	0.35	Trizol LS	N/A	qRT-PCR	3	[79]
miRs-133,-328	AMI	Plasma	EDTA	0.5-1	Trizol LS	U6 snRNA	qRT-PCR	3	[80]
miRs-17,-92a,-126,-145,-155,-199a	CAD	Plasma Serum	EDTA	0.25	TRI Reagent BD miRNAeasy	cel-miR-39	Microarray qRT-PCR	>1000	[81]
miRs-126,-133a,-499	CAD	Plasma	EDTA	U	TRI Reagent BD	cel-miR-39	qRT-PCR	7	[82]
miR-423-5p	HF	Plasma	Citrate	0.5–4.4	mirVana PARIS	miR-1249	Sequencing***; qRT-PCR	>1000	[83]
miRs-122,-126,-499	CHF	Plasma	EDTA	0.5	mirVana PARIS	Novel Synthetic miR	qRT-PCR pre-amp	3	[84]
hcmv-miR-UL112, let-7e, miR-296–5p	HT	Plasma	EDTA	0.25	TRI Reagent BD	Median normalization; U6 snRNA	Microarray qRT-PCR	>1000	[67]
miRs-27b,-130a,-210	ASO	Serum	N/A	U	QIAamp Circulating Nucleic Acid Kit	U6 snRNA	qRT-PCR	13	[85]
miR-21	AS	Plasma	EDTA	U	TRIzol	cel-miR-39	qRT-PCR	1	[86]
miRs-105,-106a,-223	FH†††	Plasma (HDL, EV)	U	U	miRNeasy	RNU6; Array Specific	Microarray qRT-PCR pre-amp	>1000	[69]‡‡‡
miRs-20a,-21,-133a, 146a,-221,-222,-210,-328	Exercise –ind. Cardiovasc. Adaptation	Plasma	EDTA	0.1	MicroRNA Extraction Kit	miR-422b	qRT-PCR	8	[87]
miRs-122,-370	HL	Plasma	EDTA	0.4	mirVana PARIS	cel-miR-39	qRT-PCR	4	[88]
miRs-1,-21,-29a,-133a,-208	MI	Plasma	U	0.4	mirVana PARIS	U6 snRNA	qRT-PCR	5	[89]
miRs-208b,-499,	MI	Plasma	Citrate	U	mirVana PARIS	cel-miRs	qRT-PCR	2	[90]
miR-133a	MI	Serum	N/A	0.1	miRNeasy	cel-miR-39	qRT-PCR	1	[91]
miRs-126,-197,-223	MI	Plasma	U	U	miRNeasy	U6 snRNA	qRT-PCR	19	[92]
miRs-15b,-27b	NSCLC	Serum	N/A	0.5	mirVana PARIS§§§	ΔCt matrix	qRT-PCR	181	[93]
miRs-1,-30d,-486,-499	NSCLC	Serum	N/A	50	TRIzol¶¶¶	Normalization to total RNA	qRT-PCR; sequencing	11 by qRT-PCR	[94]
let7f, miRs-20b,-30e-3p	NSCLC	Plama EV	U	3	Dynabeads + mirVana PARIS††	miR-142-3p and miR-30b	qRT-PCR	365	[95]
let7b, miRs-20b,-33a, 199b, 200a,-518b,-635,-662,-935	COPD, lung cancer	Whole blood	PAXgene	5	miRNeasy	Quantile normalization	Microarray; qRT-PCR	863	[96]
miRs-1, 7,-21,-30a,-126,-200b,-210,-219,-324,-451,-486	Lung cancer	Plasma	EDTA	0.2	mirVana PARIS	RNU-6B	Microarray; qRT-PCR	>200	[61]
miR-150	PAH	Plasma	Citrate	U	miRNeasy	cel-miR-39	Microarray; qRT-PCR	1223	[59]
miRs-21,-210, 486-5p	SPN	Plasma	EDTA	U	mirVana PARIS	miR-16	qRT-PCR	5	[97]
miR-483-5p	HCC	Plasma	U	0.25	miRNeasy	U6 snRNA; cel-miR-39	qRT-PCR TLDA cards A & B	5	[98]
let-7d-5p, let-7 g-5p, miRs-15b-5p,-142-3p,-191-5p,-301a-3p,-545	Alzheimer	Plasma	U	1	mirVana PARIS modified	Ath-159a; NegA	nCounter****	˜800	[99]
miR-210	AKI	Plasma	U	U	MasterPure RNA Purification Kit	cel-miR-54	Microarray	>1000; 3 by qRT-PCR	[100]

* Reagents and supplier information: Master Pure RNA Purification Kit (Epicentre Biotechnologies, Madison, WI, USA); MicroRNA Extraction Kit (BenevBio, Mission Viejo, CA, USA); miRNA Isolation Kit (RNA Bioscience, Salt Lake City, UT, USA); miRNeasy, Qiazol, QIAamp Circulating Nucleic Acid Kit (Qiagen, Valencia, CA, USA); mirVana PARIS, Trizol and Trizol LS [Ambion/Invitrogen/Applied Biosytems Inc. (ABI), Life Technologies, Carlsbad, CA, USA]; TRI Reagent BD and Tri-Reagent LS (Sigma-Aldrich, St. Louis MO, USA); Total RNA Purification Kit (Norgen Biotek Corp, Thorold, Canada).

† Quantitative real-time-PCR (qRT-PCR) technologies include TaqMan, TLDA Cards and SYBR Green methodologies using a wide range of products from ABI, Qiagen, Exiqon (Woburn, MA, USA), Roche (Indianapolis, IN, USA) and Promega (Madison, WI, USA).

‡ First study to show miRNA presence in plasma.

§ First study to analyse miRNA in plasma microvesicles in healthy individuals.

¶ Various array technologies are from Affymetrix (Santa Clara, CA, USA), Agilent (Santa Clara, CA, USA), Exiqon miRCURY LNA Array (Woburn, MA, USA), Genom Biochip MPEA (Febit biomed GmbH, Heidelberg, Germany), Fluidigm (San Francisco, CA, USA).

** Reports that some circulating miRNA species may be associated with Argonaute2.

†† Describes the use of *Caenorhabditis elegans* miRNAs spike-ins as exogenous controls for the efficiency of extraction procedure.

‡‡ Useful methods paper.

§§ Useful methods paper; compares plasma and serum.

¶¶ Comprehensive methods review paper.

*** Sequencing-based profiling from Illumnia (San Diego, CA, USA).

††† In addition to humans, study included rodent models.

‡‡‡ Report that circulating miRNAs are associated with HDL.

§§§ Reported yield 300–500 ng/ml of serum.

¶¶¶ Reported yield 5–10 μg/50 ml of serum.

**** nCounter miRNA expression assay v1 (Nanostring Technology, Seattle, WA, USA).

ACS: acute coronary syndrome; AHF: acute heart failure; AKI: acute kidney injury; AMI: acute myocardial infarction; AS: atherosclerosis; ASO: atherosclerosis obliterans; CAD: coronary artery disease; cc: cell culture; cel-miRs: *C. elegans* synthetic miR-39, miR-54 and/or miR-238; CHF: congestive heart failure; COPD: congestive obstructive pulmonary disease; DD: diastolic dysfunction; EDTA: ethylenediaminetetraacetic acid; EV: extracellular vesicles (including exosomes); FH: familial hypercholesterolaemia; H: human; HCC: hepatocellular carcinoma; HF: heart failure; HL: hyperlipidaemia; HT: hypertension; pre-amp.: pre-amplification; MI: myocardial infarction; NSCLC: non-small cell lung carcinoma; PAH: pulmonary artery hypertension; SPN: solitary pulmonary nodules; U: unknown (not mentioned in paper); VM: viral myocarditis.

However, miRNA fingerprinting as a novel and valid diagnostic, prognostic and disease surveillance tool is still in the descriptive stages. Currently, information is being gathered and compared in various disease states. The necessity of more thorough studies, based on much larger patient cohorts, is required for their utility. This is underscored by several studies using conventional biomarkers to assess tissue damage from organs that would also require invasive biopsies. For instance, comparison of circulating miRNA profile with at least one protein marker of acute coronary syndromes, namely troponin, led to conflicting results [55, 56]. At the same time, there are only few studies that analyse miRNA expression outside lung tissue in chronic obstructive pulmonary disease [57, 58], pulmonary artery hypertension [59], idiopathic pulmonary fibrosis [60] or lung cancer [61]. Several studies have begun to examine the potential of miRNAs to predict liver damage as well [62, 63].

Another level of complexity is added by the fact that, although many circulating extracellular miRNA species are repeatedly found having differential expression, such as miRs-1,-122,-126 and-223 in MI (Table 1; for comprehensive recent reviews, see Refs. [43, 64]), some of these are also associated with leukaemias and cancers [12, 66], further complicating identification of a disease-specific profile. In addition, extracellular miRNAs derived from viruses such as human cytomegalovirus can be found in patients with CVD. For example, hcmv-miR-UL112 is elevated in the circulation in hypertension [67] (Table 1). Although the role of viral miRNAs in infection is just beginning to be elucidated, little is known about their involvement in the pathogenesis of other human diseases.

Sources of variability in miRNA assessment from body fluids involve both the extraction methodology and the analysis platform employed, which may lead to inconsistent or even contradictory results. A comprehensive literature survey revealed that many methodological details are often overlooked, thus making it difficult to directly compare the specific efficiency and accuracy of various methods. The matter is further complicated by the fact that in the blood, miRNAs are either associated with proteins, such as argonaute [68], lipoproteins [69], or contained within cellular fragments designated as exosomes, microparticles, microvesicles or extracellular vesicles (EVs) [36, 37]. Extraction and analysis of miRNA from any or all of these components may pose specific challenges and yield different results.

Initial studies [36, 37] utilized a large volume of blood to isolate and profile >450 miRNAs. In contrast, advances in the approaches now allow the interrogation of <1 ml of blood to examine the expression of extracellular miRNAs (Table 1). However, in-depth analyses of the miRNA profiles often require more starting biological fluid, which may be a limiting factor. This is especially true when the patients are children, elderly or seriously ill. In addition, the RNA quantity can dictate the platform used for miRNA analysis. As shown in Table 1, current methods vary among laboratories both for miRNA isolation from plasma or serum and for obtaining miRNA profiles from these isolates.

In this review, we will highlight important considerations, such as blood collection methods or the choice of a profiling platform, when embarking on studies to profile circulating miRNAs. Throughout the review, we will refer to circulating extracellular miRNAs, as those found in the serum or plasma and not blood cells, unless otherwise stated. We focus on studies using limited sample volumes, as would be practical for disease profiling in clinical settings, and highlight optimal miRNA isolation protocols for small sample sizes. In this review, we also discuss analytic methods to identify circulating miRNAs, as well as other critical factors when designing these studies.

## Current methodologies for extracting miRNAs from plasma and serum

The purposes of detecting extracellular miRNAs are to use them as disease biomarkers, as well as to understand disease pathogenesis. Therefore, much of the effort so far involves profiling miRNA species in various body fluids to uncover differential expression patterns and to correlate them with physiological status or disease progression. The composition of these fluids is quite dissimilar, which implies that isolation methods cannot be directly transposed from one tissue/fluid to another. To date, most RNA and miRNA extraction methods use a phenol:chloroform-based extraction technique, often facilitated by adding guanidinium thiocyanate, whereas newer methods, faster and some automated, include a selective solid phase (silica) adsorption of RNA from the phenol:chloroform extraction onto mini-columns, followed by elution in water or a buffer [101]. MiRNA molecules behave physically and chemically different from the larger RNA molecules, and their quantitative recovery requires optimization of existing total RNA isolation procedures. Various manufacturers have employed different strategies for this purpose.

**Figure 1:**
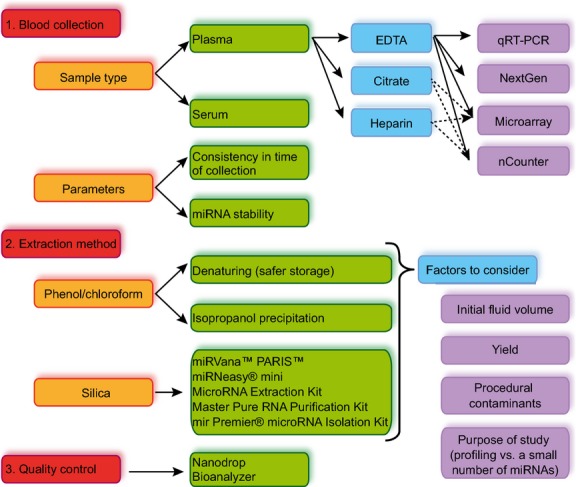
Summary of the workflow in designing miRNA profiling from plasma or serum. One needs to first decide if plasma or serum will be analysed. If plasma is selected, then the anticoagulant should be carefully chosen, because of interference with downstream applications. Consistency in the time of collection, as well as other phlebotomy parameters, is necessary, concomitant with a similar time frame of miRNA extraction. For the extraction method selection, other factors have to be considered, such as the available initial fluid volume and the expected yield. Finally, quality control is a necessary step for successful downstream applications.

MiRNA profiling is a multi-step process that includes: (*i* ) blood collection; (*ii* ) RNA purification; (*iii* ) RNA quantification and quality control; and (*iv* ) RNA profiling (Fig. 1). We will further discuss each of these steps, highlighting methodological aspects that may impact the outcome, as well as possible pitfalls. In addition to limited sample volumes, these involve: (*i* ) the lack of standard protocols; (*ii* ) interference of anticoagulants in PCR-based profiling [101]; (*iii* ) low recovery of miRNAs [64, 81]; and (*iv* ) the lack of known invariant miRNA species to be used as endogenous controls.

Lastly, we should stress the fact that it is not possible to unequivocally assign the origin of detected miRNAs. While circulating miRNAs are associated with EVs [36, 70], plasma lipoproteins [69] and proteins [68], the levels of each of these components, combined with efficiency of the extraction method used, further contribute to bias in interpreting miRNA expression levels. The sections below discuss newer methods for isolating all circulating extracellular miRNAs, regardless of plasma or serum components with which they may be associated.

### Considerations for the collection of plasma and serum

#### Plasma or serum?

The essential difference between plasma and serum is the presence and absence, respectively, of fibrinogen and clotting factors. More importantly, platelets contain a wide spectrum of miRNAs [36], and these may be released into the serum during coagulation, together with miRNAs from red and white blood cells [103]. In the clinical settings, both plasma and serum are used for extracellular miRNA detection. Plasma is routinely collected in various tubes containing anticoagulants [K_2_ ethylenediaminetetraacetic acid (EDTA), Na_3_ citrate, heparin] and serum in tubes that promote coagulation and permit clot separation from serum (serum separators). Many studies comparing these biological fluids side-by-side find little or no difference in extracellular miRNA quantification [35, 37], although higher concentrations were consistently found in sera [103].

Moreover, some important factors to consider include that: (*i* ) platelets release EVs upon stimulation [104]; (*ii* ) serum miRNAs are associated with EVs [102]; and (*iii* ) some circulating miRNAs in both plasma and serum, such as miRs-126,-150,-191 and-223, display differential expression upon platelet activation, which responds to antiplatelet therapy [105]. These variables may contribute to notable differences in reporting. Indeed, in a study dedicated to the methodological analysis of circulating extracellular miRNAs, the concentration of several miRNAs (miRs-15b,-16 and-24) was significantly higher in plasma collected in EDTA-containing tubes compared with serum [73]. Wang *et al*. found higher total miRNA concentration in serum; however, for specific miRNA, the results were more nuanced: for some of the more abundant miRNA species in blood cells (miR-150, miR-16 and miR-126), the concentrations were the same between serum and plasma, whereas for others, they observed higher concentrations in the serum (*e.g*. miR-15b and miR-451) [103].

Thus, while plasma and sera generally have similar miRNA expression patterns, in specific instances, significant differences between these biological fluids are apparent [103, 105]. As there is insufficient data to account for all of these situations, a standard approach should be followed to ensure intra-experiment or intra-clinical trial consistency. Additional caution should be used in the case of archived samples, which are mostly stored as sera [103].

#### The effect of anticoagulants

In the clinical setting, the commonly used anticoagulants for plasma collection are EDTA [36], citrate [42] or heparin [74]. In selecting a tube for blood collection, consideration must be given to the platform that will be used to profile the miRNA. One needs to avoid introducing reagents that inhibit enzymes that will be used later in the process, and that are difficult to be removed prior to subsequent profiling. For example, heparin inhibits the reverse transcriptase and polymerase enzymes used in PCR [106, 107]. Therefore, heparinase I [74] or LiCl [108] treatments of RNA preparations need to be performed prior to quantitative real-time PCR (qRT-PCR)-based profiling. Of note, removal of the heparin may not be complete and may also reduce RNA yield. Similar to heparin, citrate can interfere with PCR from blood samples [109]. Fichtlscherer *et al*. found that miRNA expression profiling was better in EDTA-anticoagulated blood compared with blood collected in sodium citrate [81]. Recently, we have confirmed that citrate interferes with qRT-PCR-based miRNA assessment [101].

In conclusion, the choice among regularly used anticoagulants should be EDTA. Citrate and heparin should be avoided as a result of their inhibitory effects on qRT-PCR. In addition, heparinized blood should be avoided for next-generation sequencing (NGS) studies as well, as these also utilize reverse transcriptase (reviewed in Section 3.5). The effect of citrate on this platform is unknown at this time. Furthermore, if a patient has several tubes of blood collected for various clinical tests, the EDTA-containing tube must be collected prior to those containing heparin or citrate, to avoid inadvertent contamination with these anticoagulants. All these critical elements need to be outlined in clinical protocols as well as in the study reports.

#### The effect of fasting and blood draw timing

Some blood components, such as glucose and lipoproteins, vary according to food intake. This is common knowledge; however, much less is known about how this impacts miRNA levels. Fasting may affect miRNA detection in different ways. First, various miRNAs may be transported in circulation as ‘cargo' by different carriers, which might themselves be affected by the fasting state of the individual. For example, HDL particles, which can carry some extracellular miRNAs, vary diurnally in their blood concentrations [69]. Second, higher levels of circulating lipoproteins might interfere with the extraction process, affecting efficiency of miRNA recovery [75]. In this context, one needs to correlate the observed changes in miRNA expression patterns in patients with atherosclerosis with their lipoprotein levels (Table 1). In the clinical setting, the general practice is to draw blood in fasting conditions; however, this is rarely mentioned in reports on circulating plasma or serum miRNAs. Wang *et al*. are one of the few research groups who have recorded this information [77].

Another factor that is not routinely considered is the diurnal variation in circulating miRNAs levels. Although it is not known whether circulating miRNAs undergo daily variations in concentration in human cases, there is at least one report of miRNA circadian rhythm in mouse serum [110]. Previously, we found no difference in the expression of extracellular miRNAs from plasma collected in the morning or afternoon from healthy individuals [36].

Thus, a good practice should be to maintain consistency in time of day for sample collection to minimize variability because of such unknown factors. Moreover, reports should attempt to capture all this information when describing their protocols in publications. Ultimately, this information will be necessary to facilitate standardization of practices for analysis of circulating miRNAs.

#### MiRNA stability and interference of blood cells with detection

miRNAs are desirable candidate biomarkers because of their stability over time, even after repeated freeze-thaw cycles in plasma and serum [35, 37, 72, 73]. Their stability is explained by several protective mechanisms, related to the association of extracellular miRNAs with plasma proteins [111], specifically argonaute-2 [68] or lipoproteins [69], or, in other circumstances, their encapsulation in EVs [36, 70]. Circulating EVs, which are phospholipid-enclosed vesicles of various sizes and cellular origins [112], deserve special attention because they are produced by many cell types, and their quantity, quality and composition vary according to the physio-pathological status, including cardiovascular events [113, 114]. One needs to take in account that the EVs are a mixed population in the blood. There are numerous published methods and several excellent reviews for EVs isolation and characterization from plasma or serum [115, 118]. To date, the technology is not adequate or advanced enough to isolate EV subpopulations of sufficient purity for miRNA profiling.

Nevertheless, although the extracellular miRNAs are stable in purified plasma or serum, it is recommended that the blood be processed within 6 hrs in EDTA tubes [119]. It is possible that the cellular components of blood are releasing miRNAs during the storage period that would account for some changes. In fact, extracellular miRNA expression changes in packed red blood cells after long-term storage [120, 121]. Thus, rapid processing, within 2–4 hrs from collection, is optimal. Furthermore, as peripheral blood cells, including erythrocytes, can contribute to extracellular miRNAs found in plasma or serum [122], increased blood cell counts in an individual need to be considered in the total miRNA recovered from blood-based analysis.

Phlebotomy variables such as lysis of erythrocytes and other blood cells, and subsequent miRNA release should be also considered [73, 123]. The importance of these factors also lies in the fact that haemoglobin and lactoferrin may be released in the process, and these have been demonstrated to inhibit subsequent qRT-PCR [107]. As discussed, platelet-derived miRNAs are present in plasma and serum [36, 105]. In some medical conditions, platelet counts increase and may be reflected as changes in plasma or serum miRNA expression patterns [124]. Although it is difficult to determine the contribution of each of these blood cell components to miRNAs found in the serum or plasma, studies should include at least WBC and platelet counts from individuals, and data should be expressed with regard to these variables. In addition, platelets can be inadvertently activated and thus induced to release miRNAs when using small gauge needles to collect blood [118]. Thus, standardization and reporting of these specific details are important when analysing circulating miRNAs as biomarkers.

In conclusion, to reduce the influence of all these factors, the protocols should be consistent in sample acquisition, storage and processing within the first 2–4 hrs from collection. Moreover, samples should be checked for small clots and haemolysis in both plasma and sera, as these could contribute to the variability in miRNAs detection. Another useful practice is to discard the first couple of ml of blood, which would remove possible tissue and cell contaminants derived from the puncture site [118]. Presentation of detailed information in publications will certainly advance our understanding of extracellular miRNAs in the circulation and how to best use them as biomarkers.

### Extraction methods

As discussed in the introduction to this section, most of present techniques for miRNA isolation are based on phenol:chloroform extraction. However, until recently, these methods were designed for use with tissues or cell cultures. A major difficulty in RNA/miRNA isolation from body fluids, including plasma and serum, is the generation of large volumes of aqueous phase. To ensure adequate denaturing and removal of the high protein content from samples (albumin, immunoglobulins, coagulation and complement components among others), the lysis reagent-to-specimen ratio has to be increased several-fold. Together with the starting fluid volume, this is the most variable step in the protocols reviewed (Table 1). Unfortunately, very few authors reported the yield of RNA recovered in the specific conditions used, making it difficult to determine the efficiency of these extraction protocols.

The transport conditions in the blood for a particular miRNA species such as argonaute-2 [68], lipoproteins [69] or EVs [36, 102] also need to be considered prior to RNA isolation. As some EVs are produced from the exocytic pathway, their membranes have a different lipid composition from cellular plasma membranes [125, 126] indicating that methods used to extract miRNAs from cells may not be suitable for extracting EVs-derived miRNAs leading to changes in expression. Indeed, comparison of RNA extraction methods from *in vitro–*generated exosomes reported that phenol-based methods were less efficient than column-based ones for total RNA [101, 127]. Conversely, a better yield of small RNAs was recovered from exosomes using combined phenol and column-based methods [101, 127]. Of particular note, the total RNA extracted from EVs consists of various RNA species, of which miRNAs comprise a small percentage and transfer RNAs are the most abundant [128]. How each method impacts the isolation of various RNA species still needs to be empirically examined. Therefore, this section highlights general protocol considerations for isolating miRNAs from plasma or serum.

#### Phenol:Chloroform extraction for miRNA

This procedure relies on the differential solubility of cellular components in organic solvents, such as phenol, chloroform or ethanol. The main components of this protocol are phenol and guanidinium thiocyanate, mostly commonly marketed as Trizol^*®*^. Because Trizol^*®*^ denatures proteins, including RNases, samples are safe for long-term storage [129]. After phase separation, RNA is recovered by precipitation with isopropyl alcohol. As miRNAs are small, ample time is needed to recover these RNA species. Thus, it is recommend that at least an overnight precipitation at −20°C or −80°C [36], followed by longer pelleting times be used, such as a 16,000–21,000 × g centrifugation for 1 hr, at 4°C.

While Trizol^*®*^ is widely employed (Table 1), there are important considerations using this method. Recently, a paper was retracted [130], as the authors realized that there was selective loss of small RNA molecules with low GC content when using Trizol, especially when low number of cells were analysed [131]. The same was found true for some pre-miRNAs, small interfering RNA duplexes and transfer RNAs [131]. Although this study refers to miRNAs extracted from cells rather than body fluids, one should nevertheless be cautious as it is unknown if these factors alter RNA isolation from plasma or serum as well.

#### Silica-based miRNA recovery methods

Several methodologies are available, of which *miRVana*™ *PARIS*™ and *miRNeasy*^*®*^ *mini* kits are more widely used (Table 1). As the majority of studies do not report the actual yield and quality of miRNA, it makes direct comparison of these methods difficult.

The *miRVana*™ *PARIS*™ kit is a commercially available method of separating both nucleic acids and proteins. The method is unique in that it isolates native proteins and small RNAs, using a non-ionic detergent to disrupt cells prior to phenol:chloroform extraction. The miRNAs are isolated over a glass-fibre filter. Specific to the *miRVana*™ *PARIS*™ method is a two-part, sequential filtration with increasing ethanol concentrations, allowing for the collection of a highly enriched fraction of RNA molecules below 200 nucleotides. This method works very well for the isolation of miRNA from tissues, and greater than a third of the articles cited in this review have used this kit for body fluids as well, as it requires small starting fluid volumes (from <100 μl, up to 625 μl). On the basis of the lack of details in these studies (Table 1), we do not know the efficiency of miRNA recovery from body fluids.

The *miRNeasy*^*®*^ *Mini* kit uses a silica-based column system to recover miRNAs. Some groups reported that this kit leads to a two-to threefold better yield than *miRVana*™ *PARIS*™ kit [75, 81]; however, only a few studies used it for miRNA extraction from plasma/serum (Table 1) [39, 62]. The kit also utilizes phenol:chloroform to separate the miRNA from other plasma components, by the adsorption on a silica mini-column in the presence of ethanol. Notably, the binding, washing and elution steps can be automated using the Qiagen QIAcube [132], which, when available, decreases both working time and variability.

Several variations of the QIAzol™ and *miRNeasy*^*®*^ protocol exist, encompassing (*i *) different starting plasma/serum volumes, between 40 and 400 μl [38, 81] and (*ii* ) different QIAzol:fluid ratios, from 1:1 [35, 37] to 3:1 [77], 3.5:1 [62], 5:1 [68] or 10:1 [134]. As the yields are almost never reported, one should empirically determine the best QIAzol™-to-sample ratio to obtain high-quality RNA from small sample volumes for one's studies. We recently published an optimized protocol in using this approach [101].

#### Other kits used for miRNA isolation from plasma/serum samples

The *MicroRNA Extraction Kit* has been used for total and miRNA isolation from 100 μl plasma that was subsequently subjected to qRT-PCR [87]. Specifically, this study examined the expression of only eight miRNAs from the recovered RNA. miRNA is extracted by homogenization in the lysis buffer rather than phenol and chloroform. The sample is then treated with ethanol. Prior to the recovery of miRNAs, the manufacturer suggests an optional in-column DNase treatment using a NucleoPur™ spin column.

For analysis of diagnostic and prognostic values of several circulating miRNAs in acute coronary syndrome, Widera *et al*. used *MasterPure*™ *RNA Purification Kit* [43]. These investigators started from 50 μl of plasma to examine the expression of a few miRNAs by qRT-PCR. Thus, both these kits recovered sufficient and suitable RNA to examine the expression of limited miRNA species.

Instead of using phenol:chloroform and silica-based columns, the Exiqon *miRCURY*^*™*^*RNA Isolation kit* is based on a proprietary resin as a separation matrix. A recent comparison of several methods for miRNA isolation from exosomes found that this kit gave the highest yields [127]. However, to date, we did not find the *miRCURY*™ *kit* used for miRNA extraction from body fluids, although in its protocol, it specifies that it can be used with as low as 100 μl of whole blood.

Sigma-Aldrich^*®*^ markets the *mir Premier*^*®*^ *microRNA Isolation Kit* to purify miRNAs and other small RNAs without phenol and chloroform. According to the manufacturer, the proprietary lysis solution achieves three functions: RNA release, inactivation of ribonucleases, and precipitation of large RNA and genomic DNA. Similar to the majority of other kits, RNA is recovered by a silica column. To date, we did not find studies using this kit to extract miRNAs from plasma or serum.

In conclusion, the choice of the specific methodology for miRNA extraction from body fluids depends on many factors, such as (*i* ) available initial volume, (*ii* ) number of miRNA species intended to be assessed, (*iii* ) method of subsequent analysis, (*iv* ) ease of use and (*v* ) price per sample. Most of these variables are investigator/study-dependent; therefore, there is no universal solution.

### Global quantity and quality assessment of miRNAs

Several methods exist to determine the concentration and quality of purified miRNAs. Spectrophotometric analysis is one of the easiest and most common methods to determine concentration and protein or phenol contamination in RNA preparations [135]. As the absorbance of phenol (270 nm) is in close proximity to that of nucleic acids (260 nm), phenol contamination can lead to overestimation of RNA quantity. Furthermore, proteins absorb light at 280 nm, but the ratio of absorbance at 260 and 280 (260/280) estimates protein contamination in a nucleic acid solution with low sensitivity [135].

It is difficult, using this method, to discern the relative composition of the isolated miRNAs compared with total RNA or other small non-coding molecules and precursor miRNAs. As both total extracellular RNA and miRNAs are recovered, the miRNA concentration is prone to be overestimated if the total RNA is degraded during the isolation process. These issues can be reflected in unreliable profiling results [136]. As extracellular miRNAs are less than 1% of the total RNA recovered [128], their concentration is often under the detection limits of spectrophotometric devices. In this instance, it is recommended to use a fixed volume rather than a fixed miRNA amount for qRT-PCR [37, 75].

When the miRNA amount and concentration are sufficient, quality assessment of the preparation can be performed by capillary electrophoresis using the *Small RNA kit* in the Agilent Bioanalyzer. This system examines the presence of small RNAs between 6 and 150 nucleotides and using an algorithm based on ribosomal RNA detection, assigns an RNA integrity number (RIN) to demonstrate miRNA quality [137, 138]. Although this system can quantify small RNA molecules <150 nucleotides, it cannot distinguish between precursor and mature miRNA forms. A low RIN sample might indicate the presence of degraded miRNA that is not of sufficient quality to profile large number of miRNAs. However, examination of individual miRNAs by qRT-PCR might still be accomplished [137]. We [36] and others [139, 140] found that RINs >6 are acceptable for miRNA profiling from plasma or serum samples. Of note, when miRNA is extracted from purified EVs, this algorithm is not applicable, as it relies on ribosomal RNAs, which are not consistent in EVs [127]. Thus, if using RIN for quality evaluation, then each investigator must determine and define the acceptable quality levels through their study.

## MiRNAs profiling methods

**Figure 2:**
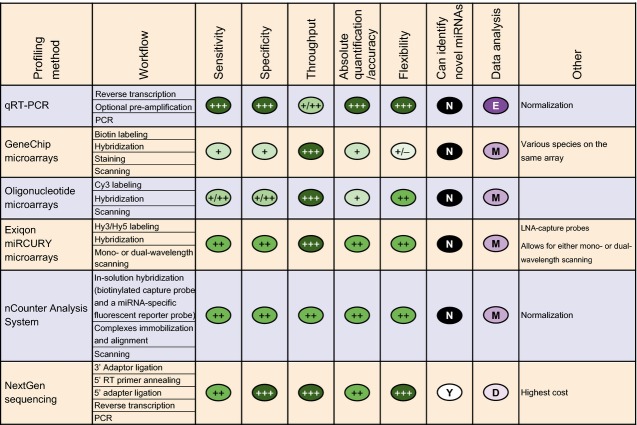
Comparison of the common miRNA profiling methods. Workflow: indicates main steps after miRNA isolation for getting the raw expression data. Sensitivity, Specificity, Throughput, Absolute quantification/accuracy and Flexibility are classified as follows: +++ (very high); ++ (moderate); +/++ (moderate to low); + (low); and +/-(low or not applicable). Flexibility: refers to ease of customization. The only technology that allows absolute quantification is qRT-PCR, whereas NextGen sequencing is the only platform that can identify novel miRNAs. Data analysis is classified as: E (relatively easy); M (moderate) with various software applications available; and D (difficult) requiring advanced computational infrastructure. Other: Considerations and challenges for the respective technologies.

To date, there are several methods to examine miRNA expression profiling including (*i* ) qRT-PCR, (*ii* ) microarrays, (*iii* ) sequence-specific hybridization in solution followed by miRNA molecules counting based on reporter probes and (*iv*) direct sequencing (Table 1). Each method has advantages and limitations (Fig. 2). In addition, miRNA assessment is also confronted with (*i* ) the inability to distinguish between precursor and mature forms of miRNAs on some profiling platforms and (*ii* ) the difficulty to validate and correlate individual miRNA expression between different profiling platforms [18, 103]. In addition, considerations for endogenous controls are dependent on the platform used. Although there are inconsistencies across profiling platforms, qRT-PCR platforms seem to have better sensitivity than array technologies for miRNA profiling from body fluids [141]. These issues and methods are briefly described below.

Using a qRT-PCR approach is limited in the species as well as the number of detectable miRNA species compared with microarrays. However, medium-and large-scale, high-throughput platforms are commercially available using a qRT-PCR approach, such as microfluidic cards or plates. These are customizable. However, for profiling a large number of miRNA species, the microfluidic cards are at the same time more flexible, but more limited in the number of miRNAs that can be examined compared with microarrays. To date, Qiagen PCR arrays cover human, mouse and rat, with primer assays extending to Rhesus, hamster, chicken, several viruses, *etc*., whereas Life Technologies assays cover ˜200 species. The design of PCR arrays can accommodate as few or as many miRNA species desired, but the higher their number, the more labour-intensive the study becomes, and less price-efficient compared with microarray methodology.

### Real-time PCR

#### Overview of qRT-PCR platforms

Analysis of miRNA expression by qRT-PCR can be performed by either TaqMan® or SYBR® Green methodologies. However, each requires specific reverse transcription (RT) and PCR reagents. For the Life Technologies miRNA TaqMan® assays, a unique stem-loop RT primer is designed to quantify only mature miRNAs and is miRNA specific. This primer produces a primer/mature miRNA chimera that extends the 3′ end of the miRNA, generating a longer RT product for the specific primers and probe to anneal during the PCR step.

Similar to the TaqMan® assay, the Qiagen SYBR® green–based *miScript PCR System* enables the profiling of several hundred miRNAs from the same sample. The RT for this system utilizes poly(A) polymerase and a unique universal oligo(dT) primer, which extends the template and converts mature miRNA species, as well as all other RNA species (precursor miRNA, other ncRNAs and mRNAs) into cDNA. The specificity is facilitated by a dual buffer system for the RT step, which selectively converts either miRNA or mRNA to cDNA. For PCR, a miRNA-specific forward primer and a universal reverse primer are used in combination with *QuantiTect SYBR Green PCR Master Mix*.

Exiqon also offers a SYBR green–based qRT-PCR system, *miRCURY LNA*™ *PCR Kit*. This kit examines the expression of individual miRNAs requiring as little as 1 pg total RNA. To increase sensitivity, miRNA-specific PCR primers generated with their ‘locked nucleic acid' (LNA™) technology [21] are utilized. The ribose rings of nucleotides are ‘locked' by connecting the 2′-O to the 4′-C atom using a methylene bridge. This locked conformation increases thermal stability of the nucleotide and high-affinity base pairing to the complementary DNA strand. Furthermore, the oligonucleotides used for PCR can be shorter and still maintain high melting temperatures.

#### Pre-amplification for qRT-PCR

To profile hundreds of differentially expressed miRNAs from serum or plasma when RNA quantity is limited poses a technical challenge. Pre-amplification of cDNA can overcome this issue. Applied Biosystems offers a kit for pre-amplification of the cDNA obtained after RT using the Megaplex™ Primer pools [36, 72]. These stem-looped RT primers reduce the number of RT reactions, as well as the amount of total input RNA required to 1 ng per sample. Pre-amplification enhances the detection of low-expressed species and does not appear to introduce a bias in miRNA detection [143]. Furthermore, Megaplex™-generated template is versatile and can be used for individual TaqMan® miRNA Assays or TaqMan® Low Density Arrays (TLDA) Cards. The TaqMan® assay is one of the most specific and sensitive methods for miRNA profiling [141, 144], and is also one of the most widely used in our literature survey. It may be labour-intensive if using a 384-well format, but the TLDA cards and the TaqMan® Open Array® MicroRNA panels simplify the profiling process. However, additional equipment is required to use these platforms.

#### qRT-PCR normalization controls

Quantification of miRNA expression by qRT-PCR may be performed in two different ways [36, 144]. One method involves calculating the absolute quantification of the number of copies of a gene using a standard curve. This is generated for each miRNA by serial dilutions of the cDNA obtained from a known amount of template. The procedure is advantageous for diagnostic purposes, especially considering that there is no consensus on endogenous controls (*as described below*). However, it is impractical to use this method when profiling a higher number of miRNA species. It has been used occasionally, for instance for finding miRNA biomarkers for early detection of lung cancer [146].

The second method is the relative quantification: the level of expression is expressed as a ratio of the Ct values of the genes of interest to the Ct values of one or more genes, the ‘endogenous controls', considered invariant in (at least) the conditions tested including the control samples [147]. The method has been widely used for mRNA and miRNA isolated from tissues and cells, as many genes and small miRNA were found relatively constant and thus useful as endogenous controls. However, characterizing miRNA expression in plasma/serum using relative quantification has encountered a serious challenge: since their identification in these fluids, no universally invariant calibrator miRNA or any other small RNA molecule has been found to date. There are a few invariant miRNAs in solid tissues [148], but their presence and/or consistent expression in various pathological instances has not yet been demonstrated for any body fluid. This problem has three solutions so far, neither perfect.

The first solution is to spike-in exogenous, synthetic miRNA mimetics. These allow for normalization, as well as estimating the efficiency of miRNA extraction and the reverse transcription step. Mitchell *et al*. used three synthetic miRNAs corresponding to *Caenorhabditis elegans* miRNAs that do not have homologous sequences in humans: cel-miR-39, cel-miR-54 and cel-miR-238 [37]. These miRNAs are spiked-in after the addition of the denaturing agents to avoid their degradation by plasma RNases. Some investigators use all three [70, 73, 74, 78], while others use only one [43, 72, 81, 82]. Finally, some groups designed their own spike-in controls [41, 84] or used synthetic human miR-422b as it is minimally expressed in plasma [87]. However, a serious drawback of the spike-in method is its reliability for normalization when quantification of extracted RNA is not possible. Spike-in miRNAs are a good measure of the extraction efficiency; however, when the starting volume is low and the amount of miRNA cannot be reliably detected, some investigators chose to use the same starting sample volume for each sample, rather than the same miRNA amount [e.g. [42, 74]]. Moreover, using a fixed volume does not imply that RNA content is invariable; therefore, spiking-in of a fixed amount of one or several synthetic miRNAs into samples containing variables amounts of RNA with the aim of normalizing qRT-PCR is not the best choice.

The second solution is to use endogenous controls, such as small nuclear (sn)RNA and small nucleolar (sno)RNAs [63, 67, 80, 85, 149], or specific miRNAs including miR-1249 [83], miR-223 or miR-16 [75]. However, these miRNAs have been shown to be invariant only in specific instances, whereas in other situations, they were found to change with disease [150]. Furthermore, snRNAs/snoRNAs are variable among tissues [151]. We have utilized snRNAs and snoRNAs as well as 18s and 5s rRNAs for normalization of plasma miRNAs [36, 70].

Finally, mean normalization of the miRNA profiling data sets is another possibility to quantify miRNA expression [36, 70]. In this case, one determines which miRNA Ct values are invariant across all (control and disease) samples. Thus, either one or a set or several consistent miRNAs across the samples can be used for normalization. One caveat is that it may be difficult to compare data using this method with other normalization methods or data representing another disease condition. More studies are needed to identify miRNAs that can serve as a true universal miRNA endogenous controls [152]. As extracellular RNA expression data are shared and placed in repositories such as Vesiclepedia [153], the identification of invariant miRNAs in body fluids should emerge.

Some general rules regarding whether to use an endogenous or exogenous control need to be considered. Either control type needs to be amendable for the miRNA assay. They should have sizes similar to miRNAs, such as snRNAs, and the amplification efficiency should be comparable to the tested miRNAs. For an endogenous control, the level of expression should be invariant across tissues and cell types as well as in various physiological and pathological conditions. Exogenous controls need to be stable. Applied Biosystems offers detection assays for several snRNAs/snoRNAs, miRNAs as well as traditional controls (18S rRNA). However, their applicability to plasma or serum samples is reduced because the presence and the variability (or lack thereof) of these small RNA species are not comprehensively established. To date, no universal endogenous control for all experimental conditions exists, neither for RNA nor miRNA, a fact especially true for body fluids including plasma and serum. Thus, selection of several (at least three) exogenous and endogenous controls with the lowest variability among all samples within a specific study should be considered for normalization.

### Hybridization-based detection of miRNA expression

A remarkable advantage of microarrays is their comprehensive coverage and, for some, the ability to be customized, thus making them a flexible and versatile tool [154]. However, specific instrumentation and software is required to perform these analyses. Depending on the microarray manufacturer, they differ according to the chemistries involved in miRNA labelling as well as probe design and methods used to immobilize the probes [155]. Moreover, as opposed to qRT-PCR, they cannot be used for absolute quantification and they have a lower sensitivity and specificity than qRT-PCR. For instance, Jensen *et al*. [141] have found that the GeneChip miRNA 2.0 Array platform from Affymetrix was not reliable at a low input level, similar to that of the miRNAs that can be recovered from small amounts of plasma (250 μl). Another limiting factor for microarrays is that, like qRT-PCR, they can only detect known miRNA species [155].

Furthermore, although both intra-and inter-platform reproducibility of data is obviously desirable, this is not always attainable. Various studies have been designed to understand the reproducibility, sensitivity and specificity of various platforms for mRNA [156] or miRNA [157]. With respect to miRNA, the results are staggering: the intra-platform reproducibility is usually good to very good; however, the agreement between various microarray technologies is low, especially for the lower expressed miRNAs [143, 157–159]. This outcome was the same regardless of the profiling technology, and qRT-PCR always proved to be the most sensitive. The inability to validate miRNA expression profiles across platforms may be due not only to differences in technologies but may also be a reflection of the lack of standard methods for (*i* ) normalization, (*ii* ) miRNA processing and (*iii* ) distinguishing mature miRNAs from precursor miRNAs or other ncRNAs.

The specific choice of microarray platform depends on several factors, an important one being availability. Many are highly sophisticated and mostly accessible in core laboratories because of the high cost of instrumentation and technical specialization. Other factors may also influence the platform choice: (*i* ) As most of the arrays require as low as 100 ng starting material, the amount of biological material available may be a deciding factor. The miRNA needs to be concentrated within a maximum volume of 3–8 μl for many platforms. This can be easily attained when the sources are tissues or cells, but much more difficult from the small sample volumes from plasma. (*ii* ) The number of miRNAs to be examined and whether the investigator is interested in, for example, viral miRNA expression indicate that a wider coverage is needed (such as Affymetrix). If the objective is to follow a specific set of miRNAs as biomarkers or to better understand their role, then arrays that encompass a lower number of miRNAs are more suitable. Some of the technologies described are customizable (*e.g*. Agilent or NanoString), whereas for others, the fabrication process makes customization less easy to apply and more expensive (*e.g*. Affymetrix). (*iii*) Finally, other important issues are sensitivity, reproducibility and a good understanding of the type of information obtained by the raw data. Below, we highlight several microarray types that are widely used and one unique system involving miRNA hybridization in fluid rather than on a solid surface.

#### Agilent oligonucleotides microarrays

Samples containing as low as 100 ng of miRNA can be profiled using the Agilent printed DNA oligonucleotide microarrays [160, 161]. The labelling method starts from total RNA, without fractionation or amplification, and consists of RNA dephosphorylation followed by dye (Cyanine 3-pCp, Cy3) ligation using T4 RNA ligase. The dynamic range is reported to be from 0.2 amol to 2 fmol miRNA, and the probe design helps to equalize melting temperatures by adding a G to the 5′ end to complement the 3′ cytosine introduced during labelling and ultimately stabilizes the cognate miRNA [161]. The slide printing allows for customizations, for instance [162], that vary mainly in the oligonucleotide probe design and miRNA labelling, as well as other procedural aspects. A good agreement was found between the detection by Agilent microarray and qRT-PCR, although a few miRNAs were consistently differentially expressed between platforms [163]. As such, this finding calls for caution when qRT-PCR is used to validate for microarray results.

#### Affymetrix GeneChip© miRNA arrays

Affymetrix is widely known for mRNA GeneChips© and has recently released GeneChip miRNA 3.0 Array. This new version covers 100% of the miRNAs described in miRBase v.17 for 153 organisms as well as viral miRNAs, all on the same array. It has probe sets for small nucleolar snoRNAs and pre-miRNA hairpins. For detection, a poly(A) tail is added to the 3′ end, then a 3DNA® dendrimer (a branched structure of single-and double-stranded DNA conjugated with numerous biotin molecules) is ligated. The biotin-labelled miRNA is then hybridized to the chip and detected by streptavidin-phycoerythrin. The miRNA GeneChip© requires a minimum of 100 ng input total RNA and is not customizable. The procedure is straightforward and has the advantage of detecting human and viral miRNAs at the same time, which may shed light into the possible connections between some diseases and various viral infections. However, several reports found low correlation between Affymetrix miRNA GeneChip results and other types of microarrays or qRT-PCR [163, 164].

### Exiqon miRCURY LNA™ microRNA arrays

This array has extensive coverage for human, mouse and rat miRNAs based on miRBase v.19 and contains 3100 capture probes as well as 146 viral miRNAs. Notably, this fluorescent system requires as little as 30 ng of input RNA. The capture probes have been designed using their LNA™ technology [21]. The LNA incorporation into the probes standardizes hybridization conditions, which are optimized against the melting temperature rather than GC content to increase specificity. This system also includes 52 spike-in control miRNAs for normalization. As for the other microarrays, the miRNA is first dephosphorylated and then a fluorophore is attached to the 3′ end. An advantage of these arrays is that they also allow for either one-or two-colour assays. However, although Exiqon claims higher sensitivity and specificity of the LNA technology, especially applicable to plasma or serum [165], we could find only few instances of its utilization for plasma miRNA profiling in humans [e.g. [166]].

### NanoString technologies nCounter© miRNA expression assay

Unlike the microarray tools, NanoString Technologies does not utilize printed chips. Rather, it uses fluorescent colour-coded ‘molecular barcode' oligonucleotides that hybridize directly to the target molecules. A biotinylated capture probe and a miRNA-specific fluorescent reporter probe are hybridized to the miRNA in solution. The tripartite-hybridized molecule is affinity-purified and attached to a streptavidin-coated slide, then imaged to count each fluorescent ‘bar code' [167]. Importantly, the nCounter© Analysis System does not rely on enzymes for processing or amplification, but requires 100 ng of concentrated RNA (33 ng/μl). Therefore, when using plasma/serum-derived miRNAs, an additional concentration step is necessary. The company claims a sensitivity close to qRT-PCR; however, the number of miRNA species detectable in a single reaction is limited to ˜800, and the normalization method may affect outcome.

### Next-generation sequencing

Perhaps, there is no more promising technology for analysing miRNA profiles than NGS, also known as massively parallel sequencing. There are currently several companies offering high-throughput NGS platforms [Illumina HiSeq200 or GAIIX, ABI SOLiD and the Roche GS FLX+ (454)], although newer smaller scale NGS platforms are becoming available to be used in individual laboratories (Illumina MiSeq, Invitrogen Ion Torrent and Roche GS Junior 454). In the short term, NGS offers important advantages over other technologies, including the possibility to generate comprehensive and definitive analyses of miRNAs in samples, comprising those derived from sera and plasma [168, 169]. The primary advantage of this technology is that it does not require knowledge of target miRNAs nor does it require specific probes or primers, and therefore does not limit studies to known miRNAs. Thus, until such time as unambiguous libraries of miRNAs are generated for humans and other organisms, NGS is currently the best platform for miRNA discovery.

Next-generation sequencing also offers several additional advantages compared to microarray profiling (reviewed by Pritchard and colleagues [155]): (*i* ) NGS is extremely sensitive. ABI claims that its SOLiD system detects one miRNA copy per cell. (*ii* ) NGS provides relative expression data for small RNAs in a sample with greater dynamic range than miRNA microarrays. Next-generation sequencing platforms discern relative expression over a six to seven log-fold range, allowing relative quantification of miRNAs that are estimated to vary in abundance by four orders of magnitude. It should be noted, however, that qRT-PCR is still the only platform capable of generating absolute quantification. (*iii* ) Another specific advantage of NGS relative to microarray technology is that NGS provides sequence data, allowing the investigator to distinguish isomiRs from miRNAs that differ by a single nucleotide, including changes related to RNA editing that may influence miRNA stability [170, 171]. (*iv* ) Lastly, NGS will generate a profile of all small RNAs in a sample, including ncRNAs, such as short interfering RNA (siRNA), piwi-RNA (piRNA) and repeat-associated siRNA (rasiRNA). Depending on the source and size range of RNA analysed, NGS may also capture other non-coding small RNAs including promoter-associated small RNAs (PASRs), transcription initiation RNAs (tiRNAs), centromere repeat-associated small interacting RNAs (crasiRNAs) and telomere-specific small RNAs (tel-sRNAs) [172].

A detailed overview of NGS platforms for miRNA sequencing and sample preparation parameters is reviewed elsewhere [155]. Sample preparation is similar for all platforms. After RNA isolation and size fractionation of the small RNA population, adaptors are ligated to the ends of the RNA molecules and reverse transcription is used to generate cDNA. PCR amplification of cDNA occurs on an immobilized surface and solid-phase PCR on the Illumina platform. The Roche and ABI platforms immobilize cDNA on beads followed by emulsion PCR.

The ABI SOLiD system requires 10–500 ng of total RNA, feasible for RNA amounts isolated from plasma or serum and can generate 120 million tags per slide or 240 million per run. The Illumina small RNA protocols require 50 ng–1 μg of RNA. Illumina systems can deliver 3.4 million to 3 billion reads per run and offer the advantage of multiplexing, which can generate 1–600 Gb of output. Sequences are then mapped to the appropriate reference genomes.

There are also some disadvantages of NGS platforms that require consideration: First, there is the high cost of profiling miRNA populations using NGS. Second, the data analysis requires an advanced computational infrastructure and bioinformatics support. We previously described a pipeline for bioinformatics analysis based on NGS data from cells infected with human cytomegalovirus including sequence tag alignment, threshold determination, visualization to reference genomes and comparison to sequences in miRBase [173]. Finally, it has been observed that miRNA sequences generated by NGS vary from miRBase reference sequence as a result of RNA editing [174, 175], 3′ end nucleotide additions and clusters of isomiRs showing variable 3′ and 5′ ends relative to the reference genome, which can influence comparison to validated sequences in miRBase.

The most profound advantage to the NGS approach is the potential for discovery of novel small RNAs such as the detection of ncRNAs. Notably, validation of these observations using experimental approaches (PCR, cloning and sequencing and/or northern analysis) to satisfy accepted miRNA criteria including size and the formation of hairpin precursor structures is necessary. Although miRBase provides a comprehensive database of validated miRNAs from multiple organisms, databases of other small RNAs are absent or still evolving. An excellent resource in this regard is RNAdb 2.0—a database of mammalian ncRNAs (http://research.imb.uq.edu.au/rnadb/) [177]. Thus, experimentally validated RNA sequences identified through NGS that do not fulfil standard miRNA criteria may require additional studies to establish classification in other small RNA families. In summary, NGS also offers researchers the opportunity to extend analysis to the exciting and rapidly expanding arena of small regulatory RNAs that may be relevant to disease. Cost and availability of computational infrastructure and bioinformatics support are top considerations when choosing this approach.

## Discussion

The search for useful diagnostic and prognostic biomarkers obtained from minimally invasive techniques is at the forefront of many disease-oriented studies. From these studies, circulating extracellular miRNAs from the blood are emerging as ideal candidates. As highlighted throughout this review, using extracellular miRNAs from body fluids as biomarkers is in its infancy. While an abundance of studies report miRNAs differential expression, important procedural information is lacking and needs to be standardized and disseminated to the research community. Importantly, attention to the fasting status of the patient and blood collection procedures, such as anticoagulants and needle size, should be considered when profiling extracellular miRNA from blood. Furthermore, extraction methods vary among reports, while information on the starting fluid volume and especially the efficiency of extraction is often missing. The availability of this information will enable investigators to standardize procedures and perhaps compare profiles across various patient cohorts.

As discussed, there are currently no known endogenous controls for serum or plasma miRNAs [36, 37]. While the miRNA profile of plasma and serum are comparable in general terms [35, 37], subtle changes in expression patterns may be present. Finally, optimization of RNA extraction methodologies from fluid samples should enable the analysis of a few miRNA species up to several hundred miRNAs. Ultimately, the RNA quantity and quality may dictate the platform used to obtain the extracellular miRNA profile.

## Conclusions

There is sufficient evidence to support circulating extracellular miRNAs, retrieved either from plasma or serum, as important players and/or promising biomarkers for a variety of diseases. Current studies are improving the ability to retrieve sufficient RNA for analysis and profile numerous miRNAs. However, the field still lacks consistency and standardization, and thus effort is needed to establish common practices. Furthermore, several important considerations are needed before designing studies to analyse circulating miRNAs. As this field evolves, circulating miRNA profiles from plasma or serum are still susceptible to significant technological advancement. The potential to obtain extracellular miRNA signature from body fluids collected by non-invasive means exists. In fact, the knowledge of isolating extracellular RNAs from plasma or serum can be extrapolated to other body fluids, such as saliva or urine [68, 165]. Certainly, this information will lead to the development of diagnostic tests from easily assessable biofluids. Thus, as future experimental and clinical investigations start filling the gaps of our knowledge, the power of using not only extracellular miRNAs but perhaps other RNA species will greatly expand our understanding of diseases and may lead to new exciting therapies.

## Key Points

- Introduction
- Current methodologies for extracting miRNAs from plasma and serum
  – Considerations for the collection of plasma and serum
  – Extraction methods
  – Global quantity and quality assessment of miRNAs
- MiRNA Profiling Methods
  – Real-time PCR
  – Microarray-based detection of miRNA expression
  – Exiqon miRCURY LNATM microRNA arrays
  – NanoString Technologies nCounterc miRNA Expression Assay
  – Next-generation sequencing
- Discussion
- Conclusions
